# Supplementary material for: Multi‐dataset identification of innovative feature genes and molecular mechanisms in keratoconus
Source: J Cell Mol Med. 2024 Sep 19;28(18):e70079. doi: 10.1111/jcmm.70079 (PMC11412914; doi:10.1111/jcmm.70079)
Supplement: Supplementary file 2 — Material S2. [file JCMM-28-e70079-s002.zip › 7.GSEA analysis/1.ARL11/KEGG_OXIDATIVE_PHOSPHORYLATION.html]

Details for gene set KEGG\_OXIDATIVE\_PHOSPHORYLATION[GSEA]

|  || Dataset | ARL11.group.cls#h\_versus\_l |
| Phenotype | group.cls#h\_versus\_l |
| Upregulated in class | l |
| GeneSet | KEGG\_OXIDATIVE\_PHOSPHORYLATION |
| Enrichment Score (ES) | -0.28125942 |
| Normalized Enrichment Score (NES) | -1.1868621 |
| Nominal p-value | 0.27459016 |
| FDR q-value | 1.0 |
| FWER p-Value | 0.988 |
Table: GSEA Results Summary

  

Fig 1: Enrichment plot: KEGG\_OXIDATIVE\_PHOSPHORYLATION      
 Profile of the Running ES Score & Positions of GeneSet Members on the Rank Ordered List

  

| SYMBOL | TITLE | RANK IN GENE LIST | RANK METRIC SCORE | RUNNING ES | CORE ENRICHMENT || 1 | ATP6V0A4 | na | 4084 | 0.198 | -0.0678 | No |
| 2 | ATP6V0D1 | na | 6786 | 0.117 | -0.1163 | No |
| 3 | NDUFA4L2 | na | 8377 | 0.085 | -0.1406 | No |
| 4 | ATP4B | na | 9357 | 0.070 | -0.1513 | No |
| 5 | NDUFC2 | na | 10744 | 0.051 | -0.1785 | No |
| 6 | NDUFS1 | na | 10818 | 0.050 | -0.1680 | No |
| 7 | ATP6V1B2 | na | 12446 | 0.032 | -0.2069 | No |
| 8 | ATP6AP1 | na | 12793 | 0.029 | -0.2096 | No |
| 9 | COX7A2L | na | 12931 | 0.028 | -0.2066 | No |
| 10 | ATP6V1E2 | na | 13257 | 0.025 | -0.2097 | No |
| 11 | COX6B2 | na | 13815 | 0.020 | -0.2206 | No |
| 12 | NDUFB5 | na | 13830 | 0.020 | -0.2159 | No |
| 13 | NDUFV3 | na | 13986 | 0.019 | -0.2156 | No |
| 14 | NDUFA1 | na | 14213 | 0.017 | -0.2178 | No |
| 15 | ATP6V1B1 | na | 14360 | 0.016 | -0.2179 | No |
| 16 | ATP6V0E2 | na | 14410 | 0.016 | -0.2153 | No |
| 17 | ATP6V1G3 | na | 14530 | 0.015 | -0.2150 | No |
| 18 | NDUFV1 | na | 14593 | 0.014 | -0.2132 | No |
| 19 | PPA2 | na | 14775 | 0.013 | -0.2152 | No |
| 20 | UQCRQ | na | 15016 | 0.011 | -0.2193 | No |
| 21 | NDUFS8 | na | 15042 | 0.011 | -0.2173 | No |
| 22 | ATP6V0A1 | na | 15062 | 0.011 | -0.2151 | No |
| 23 | NDUFB1 | na | 15196 | 0.010 | -0.2165 | No |
| 24 | NDUFS6 | na | 15253 | 0.009 | -0.2157 | No |
| 25 | NDUFC1 | na | 15395 | 0.008 | -0.2177 | No |
| 26 | ATP6V0B | na | 15548 | 0.007 | -0.2203 | No |
| 27 | COX6B1 | na | 15603 | 0.007 | -0.2201 | No |
| 28 | NDUFS5 | na | 15692 | 0.006 | -0.2212 | No |
| 29 | SDHC | na | 15742 | 0.005 | -0.2212 | No |
| 30 | NDUFB8 | na | 15934 | 0.004 | -0.2256 | No |
| 31 | NDUFA10 | na | 15939 | 0.004 | -0.2247 | No |
| 32 | COX17 | na | 16202 | 0.002 | -0.2317 | No |
| 33 | NDUFA5 | na | 16219 | 0.002 | -0.2316 | No |
| 34 | COX8A | na | 16384 | 0.001 | -0.2361 | No |
| 35 | UQCRC1 | na | 16414 | 0.001 | -0.2367 | No |
| 36 | NDUFA3 | na | 16449 | 0.001 | -0.2375 | No |
| 37 | NDUFS4 | na | 16514 | 0.000 | -0.2393 | No |
| 38 | NDUFS3 | na | 16549 | -0.000 | -0.2403 | No |
| 39 | NDUFB10 | na | 16676 | -0.001 | -0.2436 | No |
| 40 | COX7B | na | 16679 | -0.001 | -0.2434 | No |
| 41 | NDUFAB1 | na | 16740 | -0.002 | -0.2448 | No |
| 42 | NDUFA11 | na | 16918 | -0.003 | -0.2492 | No |
| 43 | NDUFA7 | na | 16940 | -0.003 | -0.2491 | No |
| 44 | NDUFB3 | na | 17132 | -0.004 | -0.2535 | No |
| 45 | NDUFB9 | na | 17155 | -0.004 | -0.2531 | No |
| 46 | COX5B | na | 17270 | -0.005 | -0.2551 | No |
| 47 | UQCRC2 | na | 17359 | -0.006 | -0.2561 | No |
| 48 | UQCR11 | na | 17430 | -0.006 | -0.2566 | No |
| 49 | NDUFA8 | na | 17455 | -0.006 | -0.2557 | No |
| 50 | COX7C | na | 17486 | -0.007 | -0.2548 | No |
| 51 | ATP6V1F | na | 17512 | -0.007 | -0.2538 | No |
| 52 | NDUFB4 | na | 17540 | -0.007 | -0.2528 | No |
| 53 | ATP6V0C | na | 17696 | -0.008 | -0.2552 | No |
| 54 | COX7B2 | na | 18020 | -0.010 | -0.2619 | No |
| 55 | COX7A2 | na | 18098 | -0.011 | -0.2614 | No |
| 56 | NDUFB7 | na | 18113 | -0.011 | -0.2589 | No |
| 57 | ATP6V1C1 | na | 18651 | -0.015 | -0.2707 | No |
| 58 | COX6C | na | 19016 | -0.017 | -0.2770 | Yes |
| 59 | ATP6V0E1 | na | 19061 | -0.017 | -0.2739 | Yes |
| 60 | COX6A1 | na | 19083 | -0.017 | -0.2700 | Yes |
| 61 | NDUFS7 | na | 19230 | -0.019 | -0.2696 | Yes |
| 62 | SDHA | na | 19462 | -0.020 | -0.2711 | Yes |
| 63 | UQCRH | na | 19528 | -0.021 | -0.2678 | Yes |
| 64 | NDUFA2 | na | 19631 | -0.021 | -0.2653 | Yes |
| 65 | NDUFB2 | na | 19632 | -0.021 | -0.2599 | Yes |
| 66 | NDUFA6 | na | 19673 | -0.022 | -0.2556 | Yes |
| 67 | ATP6V1D | na | 20147 | -0.025 | -0.2630 | Yes |
| 68 | NDUFA9 | na | 20241 | -0.025 | -0.2592 | Yes |
| 69 | COX4I1 | na | 20260 | -0.026 | -0.2533 | Yes |
| 70 | LHPP | na | 20419 | -0.027 | -0.2510 | Yes |
| 71 | ATP6V1G2 | na | 20600 | -0.028 | -0.2491 | Yes |
| 72 | PPA1 | na | 20611 | -0.028 | -0.2422 | Yes |
| 73 | COX11 | na | 20618 | -0.028 | -0.2352 | Yes |
| 74 | ATP6V1A | na | 20723 | -0.029 | -0.2309 | Yes |
| 75 | UQCRFS1 | na | 20848 | -0.030 | -0.2268 | Yes |
| 76 | UQCR10 | na | 21044 | -0.032 | -0.2244 | Yes |
| 77 | COX5A | na | 21246 | -0.033 | -0.2218 | Yes |
| 78 | ATP6V1G1 | na | 21288 | -0.033 | -0.2145 | Yes |
| 79 | NDUFV2 | na | 21789 | -0.038 | -0.2195 | Yes |
| 80 | SDHD | na | 22056 | -0.040 | -0.2169 | Yes |
| 81 | NDUFS2 | na | 22196 | -0.042 | -0.2104 | Yes |
| 82 | UQCRB | na | 22209 | -0.042 | -0.2001 | Yes |
| 83 | CYC1 | na | 22402 | -0.044 | -0.1945 | Yes |
| 84 | NDUFA4 | na | 22537 | -0.045 | -0.1868 | Yes |
| 85 | SDHB | na | 23559 | -0.057 | -0.2020 | Yes |
| 86 | NDUFB6 | na | 23631 | -0.058 | -0.1894 | Yes |
| 87 | ATP6V1E1 | na | 23880 | -0.061 | -0.1813 | Yes |
| 88 | ATP6V0D2 | na | 24169 | -0.065 | -0.1732 | Yes |
| 89 | ATP6V1C2 | na | 25402 | -0.081 | -0.1882 | Yes |
| 90 | COX15 | na | 25494 | -0.083 | -0.1698 | Yes |
| 91 | ATP6V1H | na | 25843 | -0.088 | -0.1576 | Yes |
| 92 | COX4I2 | na | 26565 | -0.100 | -0.1532 | Yes |
| 93 | COX10 | na | 27120 | -0.111 | -0.1411 | Yes |
| 94 | COX7A1 | na | 28636 | -0.147 | -0.1475 | Yes |
| 95 | COX6A2 | na | 30087 | -0.190 | -0.1412 | Yes |
| 96 | ATP12A | na | 33178 | -0.335 | -0.1457 | Yes |
| 97 | ATP4A | na | 33235 | -0.339 | -0.0613 | Yes |
| 98 | COX8C | na | 33951 | -0.412 | 0.0224 | Yes |
Table: GSEA details [plain text format]

  

Fig 2: KEGG\_OXIDATIVE\_PHOSPHORYLATION      
 Blue-Pink O' Gram in the Space of the Analyzed GeneSet

  

Fig 3: KEGG\_OXIDATIVE\_PHOSPHORYLATION: Random ES distribution      
 Gene set null distribution of ES for **KEGG\_OXIDATIVE\_PHOSPHORYLATION**
